# Genome Sequence of Non-O1 *Vibrio cholerae* PS15

**DOI:** 10.1128/genomeA.00227-12

**Published:** 2013-02-14

**Authors:** Sanath Kumar, Ingrid E. Lindquist, Anitha Sundararajan, Chythanya Rajanna, Jared T. Floyd, Kenneth P. Smith, Jody L. Andersen, Guixin He, Ryan M. Ayers, Judith A. Johnson, James J. Werdann, Ava A. Sandoval, Nadia M. Mojica, Faye D. Schilkey, Joann Mudge, Manuel F. Varela

**Affiliations:** a Eastern New Mexico University, Department of Biology, Portales, New Mexico, USA; b National Center for Genomic Resources, Santa Fe, New Mexico, USA; c Emerging Pathogens Institute, University of Florida, Gainesville, Florida, USA; d University of Massachusetts—Lowell, Department of Clinical Laboratory and Nutritional Sciences, Lowell, Massachusetts, USA

## Abstract

The draft genome sequence of a non-O1 *Vibrio cholerae* strain, PS15, organized into 3,512 open reading frames within a 3.9-Mb genome, was determined. The PS15 genome sequence will allow for the study of the evolution of virulence and environmental adaptation in *V. cholerae*.

## GENOME ANNOUNCEMENT

*Vibrio cholerae* is an estuarine bacterium that is free living or lives in association with crustaceans, algae, and fish (1). *V. cholerae* serogroups O1 and O139, which produce cholera toxin (CTX), cause the serious gastrointestinal disorder cholera (2). Non-O1 and non-O139 serogroups, of which there are roughly 200, generally do not produce CTX and are apparently not involved in epidemic-scale infections, though their involvement in diarrhea and extraintestinal infections is documented (3).

Here, we report the whole-genome sequence of a non-O1 *V. cholerae* strain, PS15, isolated from sediment in Puget Sound, WA (kind gift of Charles Kaysner). Purified genomic DNA was constructed into a sequencing library and was sequenced on the Illumina Genome Analyzer II platform. A total of 25,451,494 singleton 36-bp passing reads, approximately 234× coverage of the expected genome size, were assembled with a Kmer-sweep in Assembly By Short Sequences (ABySS) (4), followed by deredundification with CD-HIT (5) and merging of the assembly with Phrap (6). The final assembly includes 131 contigs that are >1 kb and captures most of the *V. cholerae* genome without redundancy (95% of reads realigned uniquely to the assembly). Structural gene prediction, functional annotation, and a comparative gene-based analysis with O1 *V. cholerae* N16961 were performed using the Rapid Annotations using Subsystems Technology (RAST) server (7).

*V. cholerae* PS15 has a 3,910,387-bp genome with a G+C content of 47.55%. We aligned our 131 contigs to the well-established *V. cholerae* O395 reference genome and uniquely placed 33 of our contigs to chromosome I and 65 of our contigs to chromosome II. The remaining 27 contigs that aligned to O395 had portions mapping to chromosomes, suggesting possible chromosomal rearrangements between the PS15 and O395 genomes. Six contigs did not align to *V. cholerae* O395 and may represent novel genomic regions or plasmid sequences. No complete plasmids were assembled, but similarities with plasmid sequences were found sporadically throughout the genome, suggesting prior plasmid integration.

A comparison of the PS15 genome with that of O1 *V. cholerae* N16961 (8) revealed the absence of about 619 genes. The majority (432 open reading frames [ORFs]) of these encode hypothetical proteins. Others include the *V. cholerae* virulence cassette containing *ctxAB* (cholera toxin), *zot* (zonula occludens toxin), *ace* (accessory cholera enterotoxin), *cep* (core-encoded pilin), and *orfU* genes, and virulence-associated genes, such as the *tcp* (toxin-coregulated protein) gene cluster, the *acfA* (accessory colonization factor) gene, and the *hig* toxin-antitoxin system. However, the *par* toxin-antitoxin system (*parA*, *parB*, and *parC*) was identified. About 245 ORFs were unique to the *V. cholerae* PS15 genome. A 57-kb *Vibrio* pathogenic island-2 (VPI-2), found in toxigenic *V. cholerae* strains (9), was not found in the PS15 genome, though two genes, sialic acid-related *nanM* and neuraminidase-encoding *nanH*, were identified. Interestingly, the non-O1 *V. cholerae* NRT36S genome contains an altered VPI-2 with an intact core *nan-nag* region (10). Further, PS15 harbors genes of *Vibrio* seventh pandemic island-II (VC0494 to VC0497).

The whole-genome sequence of *V. cholerae* PS15 will help our future studies to determine the differences in virulence gene composition and the molecular bases for differences in the physiology of substrate transport and utilization, environmental fitness, biofilm formation, and genome plasticity between O1 and non-O1 serogroups of *V. cholerae*.

### Nucleotide sequence accession numbers.

This Whole Genome Shotgun project has been deposited at DDBJ/EMBL/GenBank under the accession no. AIJR00000000. The version described in this article is the first version, AIJR01000000.
